# Environmental Tolerance of Entomopathogenic Fungi: A New Strain of *Cordyceps javanica* Isolated from a Whitefly Epizootic Versus Commercial Fungal Strains

**DOI:** 10.3390/insects11100711

**Published:** 2020-10-17

**Authors:** Shaohui Wu, Michael D. Toews, Camila Oliveira-Hofman, Robert W. Behle, Alvin M. Simmons, David I. Shapiro-Ilan

**Affiliations:** 1 Department of Entomology, University of Georgia, 2360 Rainwater Road, Tifton, GA 31793, USA; shaohui.wu@uga.edu; 2 USDA–ARS, Southeastern Fruit and Tree Nut Research Laboratory, 21 Dunbar Road, Byron, GA 31008, USA; camila.o.hofman@gmail.com; 3 USDA–ARS, National Center for Agricultural Utilization Research, Crop BioProtection Research Unit, 1815 N. University St., Peoria, IL 61604, USA; robert.behle@usda.gov; 4 USDA–ARS, U.S. Vegetable Laboratory, 2700 Savannah Highway, Charleston, SC 29414, USA; Alvin.Simmons@usda.gov

**Keywords:** entomopathogenic fungus, viability, virulence, temperature, ultraviolet radiation, whitefly, *Bemisia tabaci*

## Abstract

**Simple Summary:**

Whiteflies are significant pests of cotton and vegetables in southeastern USA. In previous studies, we isolated and identified a new strain of entomopathogenic fungus that caused epizootics among whiteflies in cotton fields of Southern Georgia, USA. The objective of this study was to test the level of tolerance of this new strain to environmental conditions as compared to commercial fungal strains. We exposed the new strain and three commercially available strains of biopesticides (BotaniGard, Met52, and PFR-97) to different temperatures and strong ultraviolet (UV) radiation before examining post-treatment viability and virulence against a common model organism for insect pathology, the greater wax moth larvae. We found that the new strain had similar levels of activity to commercial strains at moderate temperatures, but higher tolerance than PFR-97 to extremely low and high temperatures and strong UV intensity. These findings suggest that the new fungal strain has potential for commercial development as an alternative to PFR-97 for managing certain types of insect pests.

**Abstract:**

A new strain of *Cordyceps javanica* (wf GA17) was observed causing widespread epizootics among whiteflies in Southern Georgia in 2017. The tolerance of conidia to environmental factors including variable temperature and ultraviolet (UV) light was compared between this strain and three commercial strains of entomopathogenic fungi (*Metarhizium brunneum* F52, *Cordyceps fumosorosea* Apopka97, and *Beauveria bassiana* GHA). Under 10–30 °C, *C. javanica* wf GA17 responded similarly to other fungi, with the highest virulence against *Galleria mellonella* at 25 °C, followed by 20, 30, and 15 °C; lowest virulence was observed at 10 °C. At 35 °C and 40 °C, *C. javanica* wf GA17 had lower tolerance than *M. brunneum* F52 and *B. bassiana* GHA, but was superior to *C. fumosorosea* Apopka97 in conidia viability and post-treatment virulence. After exposure to −20 °C for 56 d, *C. javanica* wf GA17 exhibited lower germination than *M. brunneum* F52 and lower virulence than *M. brunneum* F52 and *B. bassiana* GHA, but higher germination and virulence than *C. fumosorosea* Apopka97. Following exposure to strong UV light, viability and virulence of all fungi were reduced with increasing exposure periods. Increased environmental tolerance of *C. javanica* wf GA17 over *C. fumosorosea* Apopka97 suggests that the new strain could have applicability for commercial pest management.

## 1. Introduction

The whitefly, *Bemisia tabaci* (Gennadius) (Hemiptera: Aleyrodidae), comprises a complex of biotypes, and infests a wide range of vegetable, ornamental, and field crops and may cause significant crop losses at high population densities [1,2]. The pest causes direct damage via feeding on the plant phloem, reducing plant vigor, and stunting plant growth; additionally, the feeding also induces indirect damage, including deposition of honeydew causing sooty mold on plant leaves and fruits that affects plant growth and crop values, and transmission of various viral diseases in plants [3,4]. An outbreak of *B. tabaci* MEAM1 (Middle East-Asia Minor 1, also known as biotype B) occurred in vegetable and cotton fields in southern Georgia in USA in 2017, during which a widespread naturally occurring epizootic of fungal pathogens was observed among whitefly populations [5]. The fungus was isolated and identified as a new strain (wf GA17) of the entomopathogenic fungus *Cordyceps javanica* (Friederichs and Bally) (formerly *Isaria javanica*, [6]), which was found to be highly virulent against *B. tabaci* MEAM1 in laboratory studies (Wu et al., unpublished).

The entomopathogenic fungi (EPF) can provide safe and effective suppression of arthropod pests. They have a broad host range and therefore some of them have been commercially produced as biocontrol agents [7]. Infection begins with attachment of conidia or blastospores to the host cuticle, followed by growth and penetration of the fungus through the host integument. The fungal growth blocks the digestive and circulation systems of the host and produces toxins leading to host death; eventually, aerial conidia are formed on the cadaver and disperse to infect new hosts [8,9]. When environmental conditions are highly favorable, horizontal transmission of the fungi can lead to epizootics [7]. Compared to synthetic insecticides, entomopathogenic fungi exhibit reduced risk of resistance development [10], potential for long-term persistence in the environment, and increased safety to humans and other non-target organisms [7].

The efficacy of EPF in the field may be affected by various ecological factors, including solar radiation (primarily shortwave ultraviolet (UV)), rain, temperature, humidity, surface chemistry, and phylloplane microbiota [7,8,9,11]. Among those factors, EPF tolerance of temperature extremes is considered to be particularly important as it affects fungal persistence and efficacy, as well as shelf-life during storage and transportation [11,12]. Additionally, tolerance to solar radiation is critical for field success as it affects the survival of propagules such as conidia following application. For example, Jaronski [11] found that conidial viability of *Beauveria bassiana* (Bals.-Criv.) Vuill. strain GHA decreased more slowly on the leaf undersides (9–11% per day) compared with the upper leaf surface without shade (47% per day). Thus, these environmental factors are important considerations when evaluating the commercial potential of EPF species and strains.

The tolerance of *C. javanica* wf GA17 to environmental factors has not been reported, but it is crucial for considerations of potential commercialization and field applications. Therefore, the objective of this project was to compare the environmental tolerance of *C. javanica* wf GA17 to three commercialized EPF strains (*Cordyceps fumosorosea* (formerly *Isaria fumosorosea*) (Wize) Apopka strain 97, *B. bassiana* strain GHA, and *Metarhizium brunneum* Petch (formerly *Metarhizium anisopliae* (Metchnikoff) Sorokin) strain F52. Specifically, we assessed the effect of environmentally relevant temperatures on the virulence of *C. javanica* wf GA17, as well as its tolerance to temperature extremes (high and low) and UV light.

## 2. Materials and Methods

### 2.1. Sources and Preparation of EPF

Four entomopathogenic fungal strains, *M. brunneum* strain F52 (Mb F52), *C. fumosorosea* strain Apopka97 (Cf Apopka97), *B. bassiana* strain GHA (Bb GHA), and the new strain of *C. javanica* wf GA17 (Cj wf GA17), were used in this study. Strains Cf Apopka97, Bb GHA, and Mb F52 were cultured from the commercial products PFR-97 (Certis USA LLC, Columbia, MD, USA), BotaniGard 22WP (Lam International Co., Butte, MT, USA), and Met52 EC (Novozymes, Salem, VA, USA), respectively. In contrast, Cj wf GA17 was isolated during the natural epizootic affecting whiteflies (*B. tabaci* MEAM1) in southern Georgia, USA, in September 2017 (GenBank accession no. MN453283 for internal transcribed spacer (ITS) locus; accession no. MN461230 for translation elongation factor (*tef*) gene sequences for identification of Cj wf GA17); it was deposited to the USDA–ARS Collection of Entomopathogenic Fungal Cultures (ARSEF) (accession no. ARSEF 14327).

All fungi were cultured in parallel on potato dextrose agar (PDA) for 7–10 days at 25 °C with a 14:10 (L: D) (14 h light: 10 h dark) photoperiod followed by storage at 4 °C for up to 2 weeks. Conidia were harvested by washing with sterile 0.05% Silwet L-77 (OSI Specialties, Inc., Charlotte, NC, USA) after gently scraping the surface with a sterilized blade. The mixtures were transferred to centrifuge tubes (50 mL) provisioned with six glass beads (6 mm) that facilitated mixing and fragmenting conidia clusters. Conidia suspensions were vigorously vortexed for 5 min until conidia clusters were broken apart and thoroughly mixed. Using a hemocytometer at 400× magnification, an average count from at least four samples was used to estimate the conidia concentration of each fungus. The fungal suspensions were serially diluted from the original concentration to 10^6^ viable conidia mL^−1^ with 0.05% Silwet L-77 as the solvent. Conidia viability was tested the day before experiments by spreading 0.1 mL suspension of 10^6^ conidia mL^−1^ in PDA with 1% yeast extract (60 mm), incubating at 25 °C for 16 h, and observing the germination rate of the first 200 conidia encountered at 400× magnification [13]. Conidia were considered germinated when the germ tubes were at least twice as long as the conidia [13]. 

### 2.2. EPF Virulence against Galleria mellonella Larvae at Environmentally Relevant Temperatures

The four fungal strains and an untreated control were tested against *G. mellonella* under six environmentally relevant temperatures (10, 15, 20, 25, 30, and 35 °C), modified based on the study of Miętkiewski et al. [14]. Each treatment by temperature combination was replicated three times with 10 insects per replicate. The fungal treatments were applied in 1 mL of 10^6^ viable conidia mL^−1^ and the untreated control received 1 mL of 0.05% Silwet L-77. All treatments were applied to 30–mL clear plastic Solo cups filled with 10 g of autoclaved soil in a loamy sand texture (84% sand, 10% silt, 6% clay; 2.8% organic matter; pH 6.1), and 0.4 mL of distilled water was added after treatment, resulting in final soil moisture of 14% (volume by weight). Immediately after application, a single last instar *G. mellonella* was added to each cup. Each cup was then covered with a tight fitting lid and placed on cafeteria trays, which were bagged with wet paper towels to maintain moisture before being placed in growth chambers maintained at 14:10 (L:D) photoperiod and randomly assigned to a given temperature. The percentage of insect morality and signs of mycosis (fungal growth on the cadavers) [15] were recorded at 7 and 14 days post inoculation (dpi). The entire experiment was conducted three times, for a total of 90 larvae being exposed to each temperature by treatment combination.

### 2.3. Effect of Low and High Temperatures on Conidia Viability and Virulence

Conidia viability and virulence of the four EPF strains were tested at low (−20 °C) and high (35 and 40 °C) temperatures. The −20 °C treatments were performed in a laboratory freezer, while the 35 °C and 40 °C replicates were tested in water baths. The EPF were exposed to different conditions for different lengths of time based on preliminary tests. At −20 °C, both conidia viability and virulence were observed at 0 and 56 days post treatment. At 35 °C, conidia viability was evaluated at 0, 2, 4, 8, 12, 24, 48, and 72 h of treatment; fungal virulence was checked at 0, 12, 24, and 48 h. At 40 °C, conidia viability was observed at 0, 0.5, 1, 2, 3, 4, 6, and 8 h and virulence was examined at 0, 1, 2, and 4 h of treatment. Tests were conducted by placing 1.5 mL of conidia suspension (10^6^ viable conidia mL^−1^ suspension in 0.05% Silwet L-77) in a 2 mL sterile Nalgene cryogenic vial (Thermo Scientific, Waltham, MA, USA) that was subsequently exposed to specified temperature treatments for different lengths of time. After treatment, the vial was vortexed for 1 min before taking samples to check conidia viability and virulence. A 0.1 mL aliquot was spread over PDA with 1% yeast extract (60 mm Petri dish) and incubated at 25 °C for 16 and 40 h to score the germination rate in separate dishes; two germination dishes were prepared for each observation. The virulence of conidia after temperature treatment was tested in Petri dishes (Thermo Scientific, Waltham, MA, USA), and an untreated control was used in parallel for each time point. An aliquot of 0.1 mL conidia suspension was applied to a 35–mm Petri dish lined with a single layer of sterile filter paper (30 mm, Whatman No. 1), and one last instar *G. mellonella* larva was added after application. The untreated control received 0.1 mL sterile 0.05% Silwet L-77 and the insect only. There were three replicates of 10 dishes per treatment. After treatment, the dishes were arranged in trays and placed in a growth chamber maintained at 25 °C (L:D, 14:10). Insect mortality and mycosis were evaluated at 7 and 14 dpi. The entire experiment was repeated twice.

### 2.4. Effect of UV Light on Conidia Viability and Virulence

Conidia suspensions of the four EPF were exposed to UV light to compare the UV tolerance of the new strain and the commercial strains. The experiment was carried out under a Labconco Purifier Class II biosafety cabinet (model 36209; Labconco, Kansas City, MI, USA) equipped with a 254 nm UV lamp [16]. A 1.5 mL conidia suspension of 10^6^ viable conidia mL^−1^ with 0.05% Silwet L-77 was placed in the center of a 100–mm Petri dish and then exposed to UV light inside the cabinet for 5 or 10 min. Three replicates were used for each fungus–time combination. All Petri dishes were randomly arranged under the UV lamp. After UV treatment, the fungal suspension was mixed by pipetting up and down with a 1–mL tip for 10 times and a 0.1 mL aliquot was spread over PDA with 1% yeast extract (60 mm Petri dish) and incubated at 25 °C for 16 and 40 h to score the germination rate in separate dishes; two germination dishes were prepared for each observation. The virulence of conidia after UV treatment was tested in Petri dishes as described previously for the temperature experiments. A complete set of fungal dishes that were not exposed to UV was also assessed in parallel to evaluate conidial germination and virulence. Insect mortality and mycosis were evaluated at 7 and 14 dpi. The entire experiment was repeated twice.

### 2.5. Data Analysis

Experimental design in all experiments was completely randomized, and responses (insect mortality, mycosis, and conidia germination) were analyzed using a two-way (temperature and fungus) factorial design under a linear mixed model analysis with multiple mean comparisons following the Tukey’s test (α = 0.05) (Proc Glimmix, SAS 9.4) [17]. Responses were comprised of continuous proportions between 0 and 1 and goodness-of-fit tests indicated that the beta distribution (Dist = Beta, Link = Logit) provided an acceptable model fit. In the test of extreme temperature conditions (−20, 35, and 40 °C) and UV light exposure on fungal virulence, the data were first adjusted for mortality in the control replicates using Abbott’s correction [18]. The half time for germination (LT_50_) at 16 h incubation for 35 and 40 °C treatment was calculated with probit analysis (Proc Probit, SAS 9.4); lack of overlap in the 95% fiducial limits (F.L.) of LT_50_ was used to indicate significant difference between fungi.

## 3. Results

### 3.1. EPF Virulence against G. mellonella Larvae at Environmentally Relevant Temperatures

**Figure 1:**
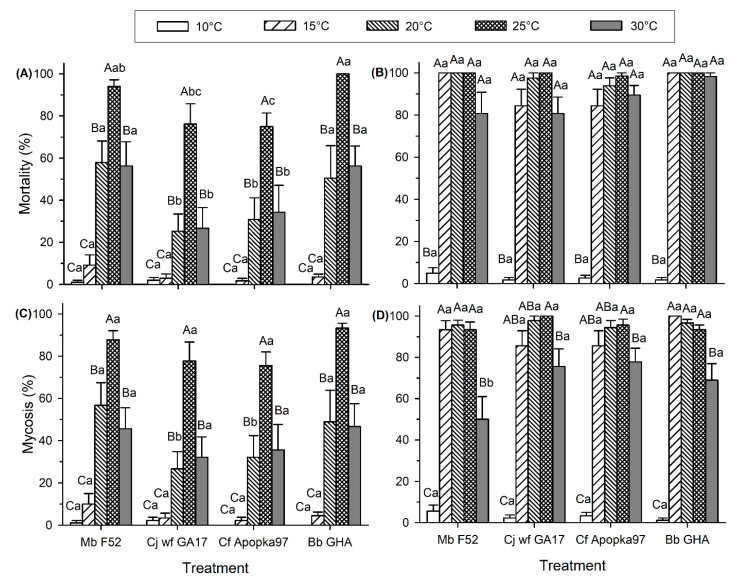
Mortality and mycosis of *Galleria mellonella* larvae exposed to *Metarhizium brunneum* strain F52 (Mb F52), *Cordyceps javanica* strain wf GA17 (Cj wf GA17), *Cordyceps fumosorosea* strain Apopka97 (Cf Apopka97), or *Beauveria bassiana* strain GHA (Bb GHA) at five temperatures in soil cups. (**A**) 7-day mortality, (**B**) 14-day mortality, (**C**) 7-day mycosis, and (**D**) 14-day mycosis. Within each subgraph, same upper-case letters indicate no significant difference among temperatures within the same fungal species, and same lower-case letters indicate no difference among fungal species within the same temperature (Tukey’s test, α = 0.05).

Virulence ranged widely by temperature. At 35 °C, control mortality was excessively high (30% at 7 dpi and 93% at 14 dpi), and no mycosis was observed in any fungal treatment. Therefore, only data observed at 10–30 °C are presented below. At 7 dpi, insect mortality was significantly affected by both temperature (F_4,158_ = 50.75, *p* < 0.0001) and fungal strain (F_3,158_ = 9.90, *p* < 0.0001), and there was no significant interaction between temperature and fungus (F_12,158_ = 1.39, *p* = 0.1761) (Figure 1A). At both 20 and 30 °C, Cj wf GA17 and Cf Apopka97 exhibited lower mortality than Mb F52 and Bb GHA; at 25 °C, Cf Apopka97 had lower mortality than Mb F52 and Bb GHA, and the mortality of Cj wf GA17 was only lower than Bb GHA. At 14 dpi, temperature had a significant effect on insect mortality (F_4,158_ = 69.67, *p* < 0.0001), while fungal strains did not affect mortality (F_3,158_ = 1.02, *p* = 0.3838); there was no significant interaction between the two factors (F_12,158_ = 0.29, *p* = 0.9906) (Figure 1B). All fungal strains showed a similar pattern that high levels of mortality were observed at 15–30 °C (close to 100%), whereas low mortality was observed at 10 °C (<5%).

Most insects that died at 7 and 14 dpi showed signs of mycosis. At 7 dpi, temperature had a significant effect on mycosis levels for each fungal species (F_4,158_ = 53.30, *p* < 0.0001), whereas fungal strain only had an effect at 20 °C; Cj wf GA17 and Cf Apopka97 exhibited lower mycosis than Mb F52 and Bb GHA (F_3,158_ = 2.67, *p* = 0.0496) (Figure 1C). There was no interaction between fungus and temperature (F_12,158_ = 0.60, *p* = 0.8410). Mycosis development followed a similar trend as mortality. At 7 dpi, all fungi had the highest mycosis at 25 °C; mycosis was lower at 20 and 30 °C, but the effects were similar. Very low mycosis occurred at 10 °C and 15 °C. At 14 dpi, mycosis levels were significantly affected by temperature (F_4,158_ = 66.91, *p* < 0.0001), but not by fungus (F_3,158_ = 1.81, *p* = 0.1478); there was a significant interaction between these factors (F_12,158_ = 2.04, *p* = 0.024) (Figure 1D). Cj wf GA17 and Cf Apopka97 had the same pattern, with the highest mycosis at 25 °C, higher than the mycosis level at 30 °C; mycosis at 15 and 20 °C was not different from that observed at either 25 or 30 °C. However, in Mb F52 and Bb GHA, similar mycosis levels occurred at 15−25 °C, significantly higher than 30 °C. Comparing across fungal strains, low mycosis occurred at 10 °C and high levels of mycoses occurred at 15−25 °C; Mb F52 had lower mycosis than other fungi at 30 °C.

### 3.2. Effect of Low and High Temperatures on Conidia Viability and Virulence

**Figure 2:**
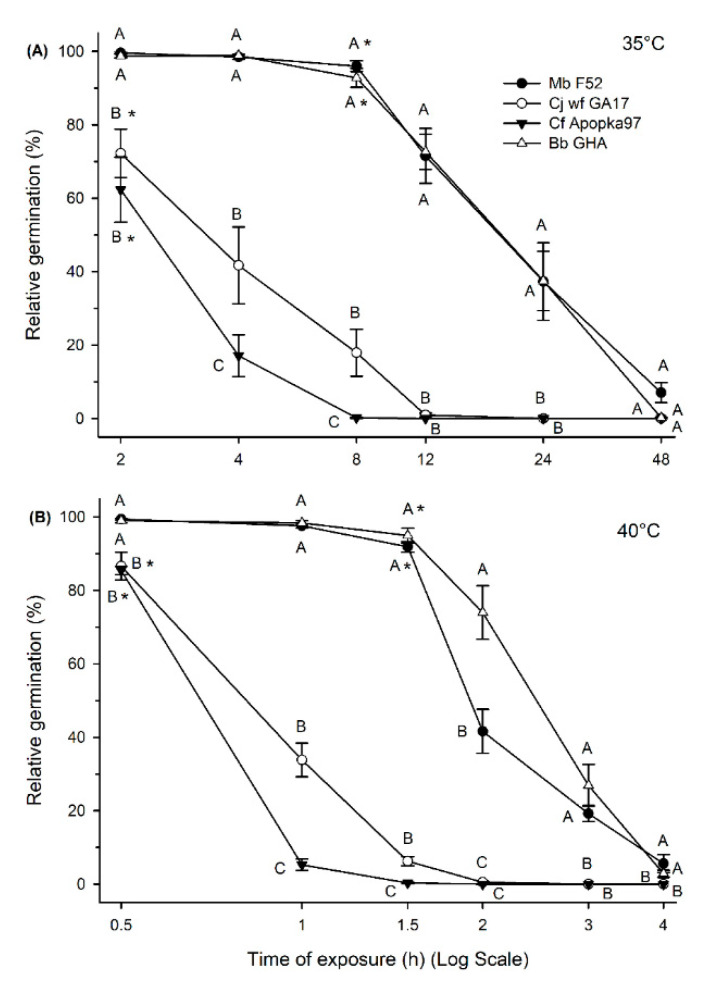
Relative germination (16 h incubation at 25 °C) of *Metarhizium brunneum* strain F52 (Mb F52), *Cordyceps javanica* strain wf GA17 (Cj wf GA17), *Cordyceps* *fumosorosea* strain Apopka97 (Cf Apopka97), or *Beauveria bassiana* strain GHA (Bb GHA) after exposure at 35 °C (**A**) and 40 °C (**B**) for various lengths of time. Within each subgraph, same letters indicate no significant difference between fungi at each time; * means the starting time point when declined germination first appeared for each fungus, comparing with control (no heat treatment).

At 35 °C, conidia viability before treatment was ≥95% for all strains. Conidia germination varied significantly due to both time of heat exposure (F_6,305_ = 106.32, *p* < 0.0001) and fungal strains (F_3,305_ = 140.21, *p* < 0.0001); there were significant interactions between time and strain (F_18,305_ = 12.89, *p* < 0.0001) (Figure 2A). A significant decline in 16 h germination appeared starting at the 8 h heat treatment for Mb F52 and Bb GHA and at 2 h for Cj wf GA17 and Cf Apopka97. Mb F52 and Bb GHA did not differ in the germination rate in all observations, which were significantly higher than Cj wf GA17 and Cf Apopka97 at all times except 48 h due to an overall low germination in all fungi. Cj wf GA17 exhibited a higher germination rate than Cf Apopka97 at 4 and 8 h heat treatment. The half time at 35 °C for 16 h germination (LT_50_) was 26.28 h (95% F.L.: 25.74–26.83 h) for Mb F52, 4.34 h (95% F.L.: 4.22–4.46 h) for Cj wf GA17, 2.72 h (95% F.L.: 2.66–2.78 h) for Cf Apopka97, and 19.49 h (95% F.L.: 19.14–19.84 h) for Bb GHA. These data suggested that Mb F52 had the highest heat tolerance, followed by Bb GHA, then Cj wf GA17, and Cf Apopka97. Delayed germination after 40 h incubation followed the same pattern as 16 h germination, and it appeared in Mb F52, Cj wf GA17, Cf Apopka97, and Bb GHA by 72 h (10.6 ± 6.3%), 24 h (1.2 ± 0.5%), 12 h (0.7 ± 0.4%), and 48 h (3.5 ± 1.1%) of heat treatment, respectively.

At 40 °C, conidia viability before treatment was >98% for all fungi. Similar to 35 °C, both time of heat treatment (F_6,139_ = 185.42, *p* < 0.0001) and fungi (F_3,139_ = 189.83, *p* < 0.0001) affected 16 h germination, and had significant interactions (F_18,139_ = 16.27, *p* < 0.0001) (Figure 2B). A significant decline in 16 h germination occurred starting at 1.5 h heat treatment for Mb F52 and Bb GHA and at 0.5 h for Cj wf GA17 and Cf Apopka97. The trend followed a similar pattern to 35 °C. Mb F52 and Bb GHA had higher germination rates than Cj wf GA17 and Cf Apopka97 in general, and Cj wf GA17 had higher germination rates than Cf Apopka97 at the 1 and 1.5 h heat treatments; however, Bb GHA had a higher germination rate than Mb F52 after 2 h exposure to 40 °C. The half time at 40 °C for 16 h germination (LT_50_) was 2.24 h (95% F.L.: 2.20–2.27 h) for Mb F52, 0.91 h (95% F.L.: 0.89–0.93 h) for Cj wf GA17, 0.70 h (95% F.L.: 0.69–0.72 h) for Cf Apopka97, and 2.52 h (95% F.L.: 2.48–2.56 h) for Bb GHA. These data suggested that, at 40 °C, Bb GHA had the highest germination rate, followed by Mb F52, then Cj wf GA17, and Cf Apopka97 still had the lowest tolerance. However, delayed germination at 40 h was observed in Mb F52, Cj wf GA17, Cf Apopka97, and Bb GHA by 8 h (12.3 ± 2.3%), 4 h (0.3 ± 0.2%), 2 h (0.3 ± 0.3%), 8 h (0.9 ± 0.5%) of heat treatment, respectively, indicating similar thermotolerance of Bb GHA and Mb F52 at 40 °C.

**Figure 3:**
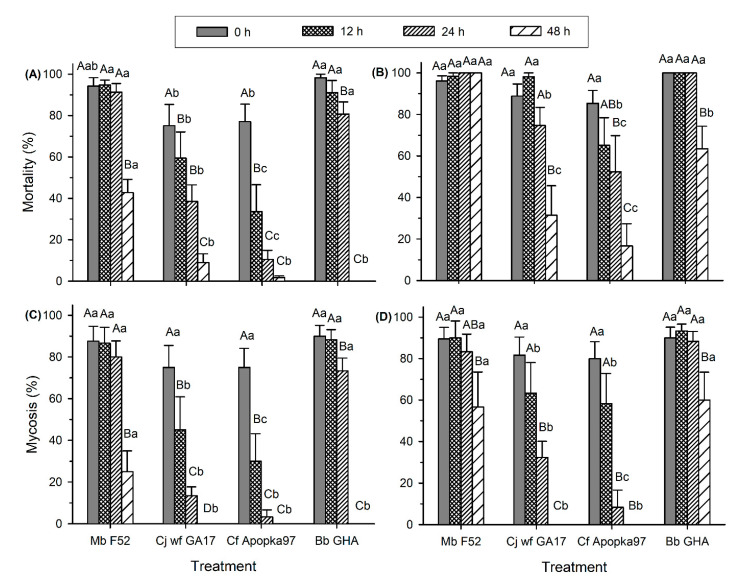
Mortality and mycosis of *Galleria mellonella* larvae exposed to *Metarhizium brunneum* strain F52 (Mb F52), *Cordyceps javanica* strain wf GA17 (Cj wf GA17), *Cordyceps fumosorosea* strain Apopka97 (Cf Apopka97), or *Beauveria bassiana* strain GHA (Bb GHA) treated at 35 °C for 0, 12, 24, and 48 h, at 7 and 14 days post inoculation (dpi). (**A**) 7-day mortality, (**B**) 14-day mortality, (**C**) 7-day mycosis, and (**D**) 14-day mycosis. Within each subgraph, same upper-case letters indicate no significant difference among time of exposure within the same fungal species, and same lower-case letters indicate no difference among fungal species within the same exposure time (Tukey’s test, α = 0.05).

After treatment at 35 °C, the fungal virulence was also affected, varying with time of heat treatment (F_3,79_ ≥ 15.26, *p* < 0.0001) and fungal strains (F_3,79_ ≥ 14.94, *p* < 0.0001), in causing both insect mortality and mycosis at both 7 and 14 dpi (Figure 3A–D). There were significant interactions between heat treatment and fungus (F_9,79_ ≥ 3.26, *p* ≤ 0.0020). At 7 dpi, decline in mortality started after 48, 12, 12, and 24 h of heat treatment for Mb F52, Cj wf GA17, Cf Apopka97, and Bb GHA, respectively (Figure 3A). Mycosis development followed a similar pattern (Figure 3C). At 7 dpi, Cj wf GA17 caused higher mortality than Cf Apopka97 at 12 and 24 h and higher mycosis at 12 h, but was lower than Mb F52 and Bb GHA. At 14 dpi, insect mortality was similarly high within 48 h heat exposure in Mb F52, while a decline in mortality occurred at 48 h in Cj wf GA17 and Bb GHA and started at 24 h in Cf Apopka97 (Figure 3B). Mycosis had a similar trend to mortality at 14 dpi, except that reduced levels appeared at 48 h for Mb F52 and at 24 h for both Cj wf GA17 and Cf Apopka97 (Figure 3D). At 14 dpi, again Cj wf GA17 caused higher mortality than Cf Apopka97 at 12 h and 24 h, but higher mycosis at 24 h, and was lower than Mb F52 and Bb GHA except for mortality after 12 h heat exposure. No mycosis occurred in Cj wf GA17 or Cf Apopka97 after 48 h treatment.

**Figure 4:**
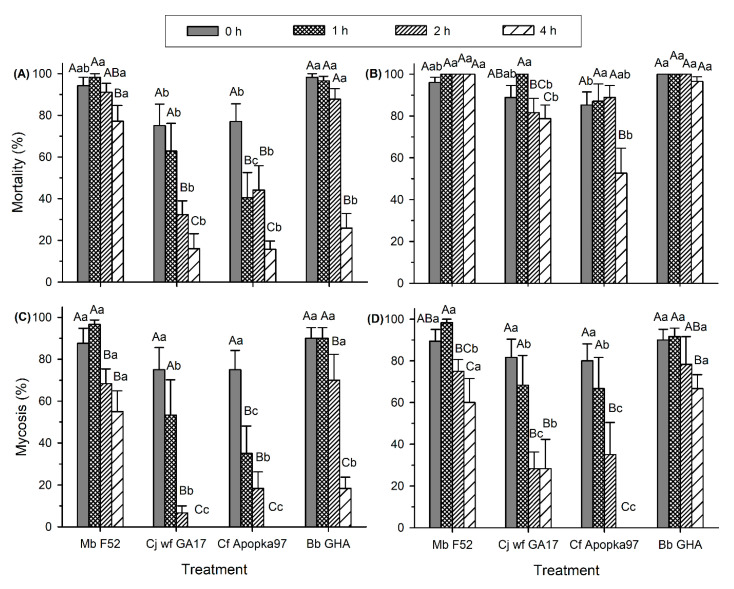
Mortality and mycosis of *Galleria mellonella* larvae exposed to *Metarhizium brunneum* strain F52 (Mb F52), *Cordyceps javanica* strain wf GA17 (Cj wf GA17), *Cordyceps fumosorosea* strain Apopka97 (Cf Apopka97), or *Beauveria bassiana* strain GHA (Bb GHA) treated at 40 °C for 0, 1, 2, and 4 h, at 7 and 14 days post inoculation (dpi). (**A**) 7-day mortality, (**B**) 14-day mortality, (**C**) 7-day mycosis, and (**D**) 14-day mycosis. Within each subgraph, same upper-case letters indicate no significant difference among time of exposure within the same fungal species, and same lower-case letters indicate no difference among fungal species within the same exposure time (Tukey’s test, α = 0.05).

Virulence after exposure to 40 °C was also affected by both time of heat exposure (F_3,79_ ≥ 6.52, *p* ≤ 0.0005) and fungal strain (F_3,79_ ≥ 10.62, *p* < 0.0001), with a significant interaction between the factors (F_9,79_ ≥ 2.28, *p* ≤ 0.0252) (Figure 4A–D). At 7 dpi, decline in mortality started after 4 h of heat exposure for Mb F52, 2 h for Cj wf GA17, 1 h for Cf Apopka97, and 4 h for Bb GHA, while reduced mycosis started after 2, 2, 1, and 2 h of heat treatment for Mb F52, Cj wf GA17, Cf Apopka97, and Bb GHA, respectively (Figure 4A,C). At 7 dpi, Cj wf GA17 showed higher mortality and mycosis than Cf Apopka97 at 1 h heat treatment, and was lower than Mb F52 in all heat treatments and lower than Bb GHA except mortality after 4 h treatment. At 14 dpi, mortality caused by Mb F52 and Bb GHA remained high after 4 h heat treatment, while Cj wf GA17 and Cf Apopka97 had reduced mortality at 4 h; decreased mycosis occurred at 4, 2, 2, and 4 h of heat treatment for Mb F52, Cj wf GA17, Cf Apopka97, and Bb GHA, respectively (Figure 4B,D). At 14 dpi, Cj wf GA17 had a similar response to Cf Apopka97, but higher mycosis, in the 4 h treatment, in which Cf Apopka97 had no fungal infection. In general, at 40 °C, fungi followed a similar pattern to 35 °C, with higher virulence of Cj wf GA17 than Cf Apopka97, but lower than Mb F52 and Bb GHA, although the duration of tolerance was shorter at 40 °C.

**Figure 5:**
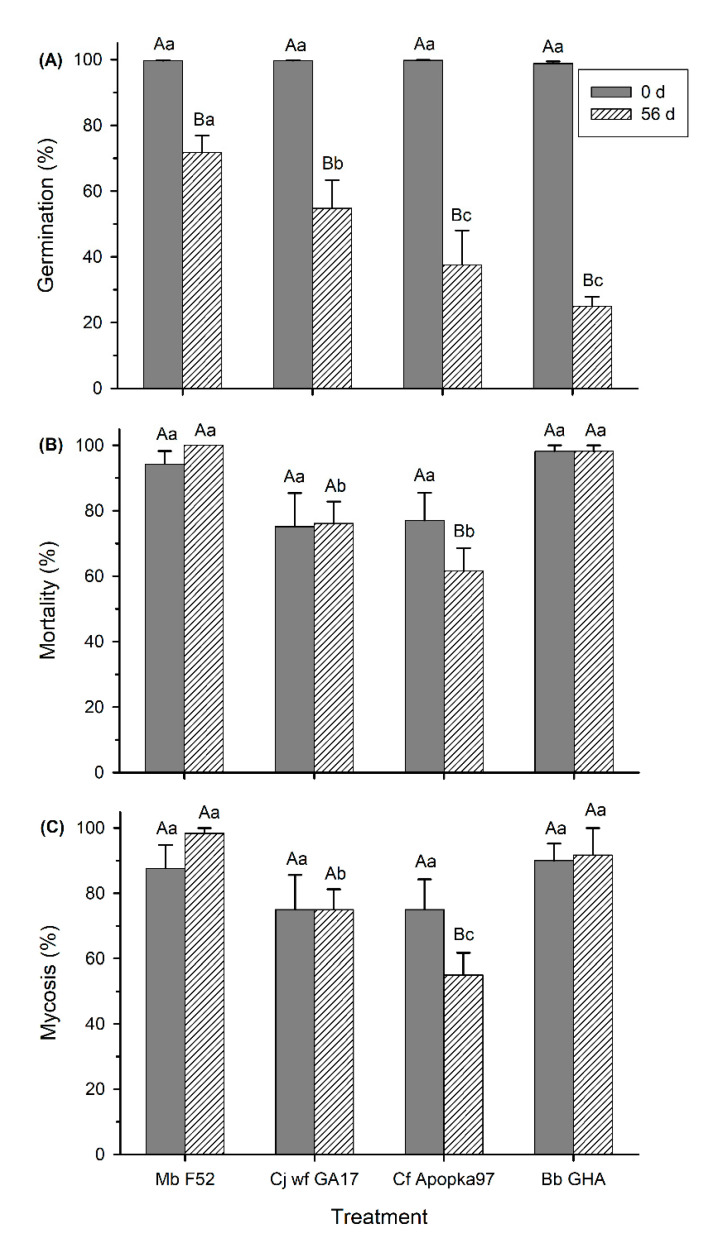
Effect of low temperature (0 and 56 d at −20 °C) on 16 h germination (**A**), mortality (**B**), and mycosis (**C**) of *Metarhizium brunneum* strain F52 (Mb F52), *Cordyceps javanica* strain wf GA17 (Cj wf GA17), *Cordyceps fumosorosea* strain Apopka97 (Cf Apopka97), or *Beauveria bassiana* strain GHA (Bb GHA) against *Galleria mellonella* larvae at 7 days post inoculation. Within each subgraph, same upper-case letters indicate no significant difference among time of exposure within the same fungal species, and same lower-case letters indicate no difference among fungal species within the same exposure time (Tukey’s test, α = 0.05).

At −20 °C, compared with day 0 (no cold treatment), the 56-day treatment had reduced germination of all fungi (F_1,39_ = 161.47, *p* < 0.0001), and the level of reduction varied with fungal strain (F_3,39_ = 3.65, *p* = 0.0206) (Figure 5A). After 56 days, Mb F52 had the highest germination, followed by Cj wf GA17, with Cf Apopka97 and Bb GHA having the lowest germination. The effects of cold treatment on fungal virulence as indicated by insect mortality and mycosis at 7 dpi varied among fungal strains (F_3,39_ ≥ 6.25, *p* ≤ 0.0014), ranging from no impact on Mb F52, Cj wf GA17, and Bb GHA, to significant suppression on Cf Apopka97 (F_1,39_ ≥ 4.85, *p* ≤ 0.0336) (Figure 5B,C).

At the 14 dpi observations, insect mortality was ≥ 85.1% and mycosis was ≥ 76.7% in all treatments; compared with 0 day, there was no treatment effect in reducing either mortality or mycosis (*p* > 0.05). Although Cf Apopka97 had lower mycosis (76.7 ± 6.1%) than other fungi (98.3 ± 1.7% in Mb F52, 88.3 ± 4.8% in Cj wf GA17, and 91.7 ± 8.3% in Bb GHA) for the 56-day exposure (F_3,39_ = 4.00, *p* = 0.0141), there was no difference between days 0 and 56 in any fungus (F_1,39_ = 1.41, *p* = 0.2422).

### 3.3. Effect of UV Light on Conidia Viability and Virulence

**Figure 6:**
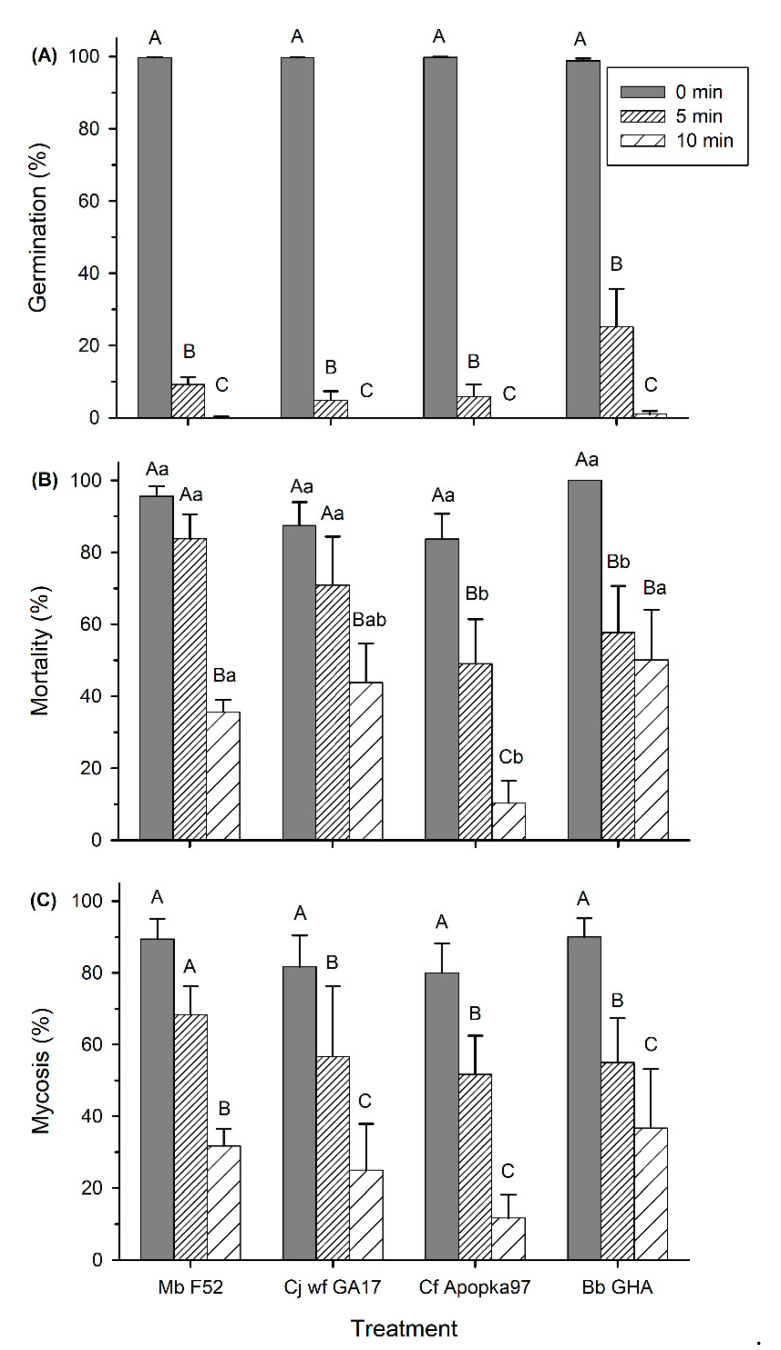
Effect of UV exposure (0, 5, or 10 min) on 40 h germination (**A**), mortality (**B**), and mycosis (**C**) of *Metarhizium brunneum* strain F52 (Mb F52), *Cordyceps javanica* strain wf GA17 (Cj wf GA17), *Cordyceps fumosorosea* strain Apopka97 (Cf Apopka97), or *Beauveria bassiana* strain GHA (Bb GHA) against *Galleria mellonella* larvae at 14 days post inoculation. Within each subgraph, same upper-case letters indicate no significant difference among time of exposure within the same fungal species, and same lower-case letters indicate no difference among fungal species within the same exposure time (Tukey’s test, α = 0.05).

Before UV treatment, conidia viability was ≥99% for all fungi. After UV exposure and subsequent 16 h of incubation at 25 °C, no germination occurred in most treatments, except for 0.7 ± 0.2% germination of Bb GHA exposed to 5 min UV. UV light significantly inhibited conidia germination of all fungi (F_2,59_ = 496.79, *p* < 0.0001); except for Bb GHA being marginally more tolerant than other fungi at 5 min UV (F_3,59_ = 2.97, *p* = 0.039), there was no differences among fungi in UV susceptibility otherwise (data not shown). After UV exposure and subsequent 40 h incubation, germination was significantly inhibited by UV exposure (F_2,59_ = 126.75, *p* < 0.0001), regardless of fungal strains (*p* > 0.05). Both 5 min and 10 min UV treatment decreased conidia germination of all fungi; the inhibition was more pronounced at 10 min, with only 0.2% germination of Mb F52 and 1.0% germination of Bb GHA and no germination of Cj wf GA17 and Cf Apopka97 (Figure 6A).

At 7 dpi, insect mortality and mycosis were very low (≤ 10%) and were significantly affected by UV treatments at both 5 min and 10 min lengths (F_2,59_ ≥ 74.47, *p* < 0.0001), regardless of fungal strain (*p* > 0.05). At 14 dpi, insect mortality was affected by both time length of UV treatment (F_2,59_ = 39.10, *p* < 0.0001) and fungal strain (F_3,59_ = 8.22, *p* = 0.0001); mycosis was affected by UV treatment (F_2,59_ = 39.84, *p* < 0.0001), but not by fungal strain (*p* > 0.05) (Figure 6B,C). There was an interaction between UV treatment and fungus-induced mortality (F_6,59_ = 2.42, *p* = 0.0372), but not in mycosis (*p* > 0.05). Compared to observations before UV treatment, a significant decline in mortality and mycosis appeared in all treatments except after 5 min exposure of Mb F52 and Cj wf GA17 (reduced mycosis). Compared to 5 min UV, the 10 min treatment resulted in lower mortality and mycosis in all fungi, except there was no difference in mortality caused by Bb GHA. For 5 min UV, Cf Apopka97 and Bb GHA had lower mortality than Mb F52 and Cj wf GA17; for 10 min, Cf Apopka97 had lower mortality than Mb F52 and Bb GHA, but was not different from Cj wf GA17 (Figure 6B). However, all fungi had similar levels of mycosis at the same UV treatment (Figure 6C).

## 4. Discussion

Under environmentally relevant temperatures (10–30 °C), virulence of Cj wf GA17 followed a similar pattern as other fungi, with the highest virulence at 25 °C, followed by 20 and 30 °C, delayed virulence at 15 °C, and low virulence at 10 °C. However, in early observations (7 dpi), Cj wf GA17 and Cf Apopka97 tended to have lower virulence than Mb F52 and Bb GHA. At 30 °C, in late observations (14 dpi), all fungi had reduced mycosis than at 25 °C; particularly, Mb F52 was lower than other fungi; at 35 °C, no fungi showed mycosis development. This was probably due to inhibition of fungal viability and virulence, and competition from saprophytic fungi and bacteria, which outgrew the entomopathogenic fungi under the high temperature. Ekesi et al. [19] found that the optimum temperature for germination, radial growth, and virulence of two strains of *B. bassiana* and four strains of *M. anisopliae* on the legume flower thrips, *Megalurothrips sjostedti*, ranged between 25 and 30 °C; activities of all tested fungal strains were low at 15 and 35 °C. In addition, Miętkiewski et al. [14] reported no infection of *B. bassiana*, *M. anisopliae*, and *Paecilomyces fumosoroseus* (*C. fumosorosea*) at 35 °C under which *G. mellonella* was exposed to pine litter as a “bait”; the highest activity of *M. anisopliae* occurred at 30 °C, *B. bassiana* at 25 °C, and activity of *P. fumosoroseus* appeared at 15–25 °C only, with optimum growth at 27.5, 25, and 25 °C, respectively. This pattern is similar to our findings, except that in our test, *M. brunneum* was less tolerant to high temperatures and had lower virulence at 30 °C than at 25 °C, which was probably due to strain difference. Cabanillas and Jones [20] found that *Isaria* sp. (*Cordyceps* sp.), which was later identified as *Isaria poprawskii* sp. nov. [21], a species closely related to *C. javanica*, did not grow at constant 35 °C for 7 days, but it had optimum growth at 30 °C. This is different from the optimum virulence at 25 °C and reduced virulence at 30 °C in our test, which suggests that the two species differ in temperature tolerance.

The strain Cj wf GA17 showed lower tolerance than Mb F52 and Bb GHA to high temperatures of 35 and 40 °C in both viability and virulence. Similarly, Souza et al. [22] found that *I. fumosorosea* (*C. fumosorosea*) strain ARSEF 3889 had lower tolerance than *M. brunneum* ARSEF 1187 and *B. bassiana* ARSEF 252, while *M. brunneum* ARSEF 1187 had lower LT_50_ of conidial germination than *B. bassiana* ARSEF 252 (1.83 versus 3.54 h) at 45 °C for 48 h germination. In our study, Mb F52 had higher LT_50_ than Bb GHA at 35 °C (26.3 versus 19.5 h), but slightly lower LT_50_ at 40 °C (2.2 versus 2.5 h). Such a difference was probably due to differences in strains and temperatures.

Superior heat tolerance in Cj wf GA17 over Cf Apopka97 was observed at the high temperatures of 35 °C and 40 °C in both conidia viability and virulence. However, these advantages may be compromised or diminished at even longer exposure to higher temperatures, given that neither of the fungi survived long-term exposure (lost viability by 24 h at 35 °C and 4 h at 40 °C or earlier). Previously, there have been several reports in delayed germination of *B. bassiana* and *M. anisopliae* in response to heat stress [23,24,25,26]. This phenomenon was also seen in all four fungi in our test; additionally, we observed that the conidia may eventually lose viability for a longer time of heat treatment. Similarly, in the study of Cabanillas and Jones [20], *Isaria* sp. (*Cordyceps* sp.) did not grow when held at a constant 35 °C for 7 days, but was able to recover and grow when transferred to 25 °C; no recovery or growth occurred at either constant 40 °C or transfer from 40 to 25 °C. This indicated that, in short-time heat stress, germination and growth of the fungus may be suppressed and retarded; for longer time or higher temperature of exposure, however, the damage may become irreversible and the fungus may lose viability permanently.

Similar to tolerance of high temperatures, Cj wf GA17 had higher viability and virulence than Cf Apopka97 and lower virulence than Bb GHA and Mb F52 at −20 °C. However, Cj wf GA17 only had lower germination than Mb F52; while Bb GHA had the lowest germination, its virulence was not affected, probably because the amount of survived conidia was adequate to infect the host. Fernandes et al. [26] found that *B. bassiana* strain ARSEF 252 was more cold-active than *Metarhizium* spp. isolates at 5 °C. The trend is different from our findings, yet this may be explained in that we used different strains and investigated lower temperatures.

The effect of UV on Cj wf GA17 virulence was similar to other fungi. Although Cj wf GA17 had higher insect mortality than Cf Apopka97 and Bb GHA after 5 min exposure, it caused similar levels of mycosis to other fungi. In contrast, Cf Apopka97 caused lower mortality than Mb F52 and Cj wf GA17 after 5 min treatment and lower than Mb F52 and Bb GHA after 10 min treatment, although the mycosis levels were not different. It is worthwhile to mention that *B. bassiana* GHA had marginally higher viability than other fungi. Fargues et al. [27] found that *B. bassiana* isolates had stronger tolerance to ultraviolet B (UV-B) (≥295 nm) than *M. anisopliae* and *I. fumosorosea* (*C. fumosorosea*) isolates; the latter isolates were most susceptible. Our results followed a similar pattern, but the intensity of UV light used (254 nm) in our treatments was too strong to more clearly separate the fungi in their UV susceptibility. The impact of solar radiation on fungal activity should be further investigated under longer wavelengths of UV.

Delayed germination after 40 h incubation appeared in all fungi exposed to UV light for 5 min. Delayed germination of EPF in response to UV light has been reported in other studies [27,28,29,30,31], and UV wavelengths in these studies belong to either ultraviolet A (UV-A) or UV-B, less intensive to that used in the current study. The delay in germination suggest that part of the damage caused by short exposure of UV may be repaired before germination occurs. However, similar to heat stress, the damage caused by UV may be permanent and irreversible given longer time of exposure. In the current study, 10 min (or longer in the preliminary test) of UV exposure killed almost all fungal conidia; when the dishes were incubated longer than 40 h, very few colony forming units appeared. The mechanism of damage repair in stress tolerance may also apply to heat stress, as delayed germination occurred at 35 and 40 °C treatment, regardless of fungi.

## 5. Conclusions

Overall, under environmentally important temperatures, Cj wf GA17 responded similarly to commercial strains, with optimum virulence at 25 °C. Fungal virulence and viability were inhibited by high temperatures. The tolerance of Cj wf GA17 to extremely low or high temperatures was inferior to Mb F52 and Bb GHA, but it was superior to Cf Apopka97. In tolerance of UV light, it had similar response to other fungi, except marginally lower viability than Bb GHA. The advantage of Cj wf GA17 over Cf Apopka97 in environmental tolerance makes it a good alternative to, or even a replacement of, Cf Apopka97 in pest management. Previously, this new strain showed higher virulence than Cf Apopka97 against *B. tabaci* (MEAM1) and a similar effect against *Aphis gossypii* in the laboratory (Wu et al., unpublished), which indicate its good potential for managing foliar-feeding pests such as whiteflies. However, weather conditions and the time of spray should be taken into account in field applications to avoid direct exposure of fungal propagules to heat and UV radiation. This study provides a valuable reference for forthcoming field studies on post-application persistence and efficacy of Cj wf GA17 as compared to Cf Apopka97 for managing whiteflies.

## 6. Patent

The United States Department of Agriculture (USDA) has passed on the patent potential of the research findings reported in this manuscript.
